# Clinical and Biological Risk Factors for Neuropsychological Impairment in Alcohol Use Disorder

**DOI:** 10.1371/journal.pone.0159616

**Published:** 2016-09-12

**Authors:** Ludivine Ritz, Laurent Coulbault, Coralie Lannuzel, Céline Boudehent, Shailendra Segobin, Francis Eustache, François Vabret, Anne Lise Pitel, Hélène Beaunieux

**Affiliations:** 1 U1077, INSERM, Caen, France; 2 UMR-S1077, Université de Caen Normandie, UMR-S1077, Caen, France; 3 UMR-S1077, Ecole Pratique des Hautes Etudes, Caen, France; 4 U1077, Centre Hospitalier Universitaire, Caen, France; 5 Centre Hospitalier Universitaire, service d’addictologie, Caen, France; 6 Centre Hospitalier Universitaire, Laboratoire de biochimie, Caen, France; 7 Université de Caen Normandie, Laboratoire EA4650, Caen, France; University of Kentucky, UNITED STATES

## Abstract

The effects of alcoholism on cognitive and motor functioning are heterogeneous. While the role of some factors (patterns of alcohol consumption, eating habits or associated liver disease) has been hypothesized, the origins of this heterogeneity remain difficult to establish. The goals of the present study were thus to identify the clinical and biological risk factors for alcohol-related neuropsychological impairments and to determine the threshold beyond which these risk factors can be considered significant. Thirty alcoholic patients and 15 healthy controls had a blood test and underwent a neuropsychological examination. Alcohol severity measures, and liver, thiamine and malnutrition variables, were included in logistic regression models to determine the risk factors for cognitive and motor impairments (executive functions, visuospatial abilities, verbal episodic memory, ataxia), as well as those related to the severity of patients’ overall neuropsychological profile (moderate or severe impairments). Liver fibrosis was found to be a risk factor for executive impairments and also for ataxia, when it was associated with long-term alcohol misuse and symptoms of withdrawal. Altered thiamine metabolism was solely predictive of verbal episodic memory impairments. This combination of biological abnormalities was associated with a profile of moderate neuropsychological impairments. Malnutrition was associated with a profile of more severe impairments. Malnutrition, altered liver function and thiamine metabolism explain, at least partially, the heterogeneity of alcohol-related neuropsychological impairments. Our findings could allow clinicians to identify patients at particular risk of severe neuropsychological impairments before the onset of irreversible and debilitating neurological complications.

## Introduction

The neuropsychological profile associated with alcohol use disorders encompasses deficits in executive functions [1], working memory [2], episodic memory [1], visuospatial abilities [3,4] and motor functions including ataxia [3]. Although these impairments are reported to concern a large proportion of alcoholic patients (AL) [1], the effects of chronic alcoholism on cognitive and motor functioning are heterogeneous [5]. According to the continuum hypothesis [6], alcohol-related neuropsychological impairments range from mild-to-moderate [3] to severe deficits [5] comparable to those observed in patients with Korsakoff’s syndrome [7], and the main contributing factor is the total amount of alcohol consumed over the drinking lifetime [6]. However, some studies have indicated that recent alcohol consumption may be a better predictor of neuropsychological performances [8,9]. Although other alcohol variables have been regarded as potential explanations for patients’ neuropsychological profiles, such as the duration of alcohol dependence [3,8], length of sobriety [9], and number of withdrawals [10], there is still no consensus as to the sources of the cognitive heterogeneity observed in AL. Other potential factors indirectly related to the pattern of chronic alcohol consumption, such as malnutrition, thiamine deficiency and liver complications, therefore need to be considered as well.

AL are at particular risk of malnutrition because of modifications to their eating habits and/or compromised absorption of nutrients in the gastrointestinal tract [11]. Malnutrition is one of the operational criteria for the in vivo diagnosis of Wernicke’s encephalopathy [12], and has been associated with mild-to-moderate cognitive impairments in AL [5]. However, relationships between malnutrition and neuropsychological deficits in AL have never been described in the literature. Decreased dietary intake and the deleterious effects of alcoholism on thiamine transport, storage and utilization [13,14] seem to be responsible for thiamine deficiency. Thiamine deficiency is found in 30-80% of AL [15], and can result in Wernicke’s encephalopathy [16] and Korsakoff’s syndrome, which is characterized by severe and irreversible neurological complications [7]. Four different forms of thiamine are present in the human body: unphosphorylated thiamine (thiamine T), thiamine monophosphate (TMP), thiamine diphosphate (TDP) and thiamine triphosphate (TTP). TDP is regarded as the physiologically active form of thiamine. Studies conducted in AL before the administration of vitamin supplements have shown that TMP [17] and TDP [18] concentrations are lower in patients than in healthy controls, whereas thiamine T concentration is abnormally high in some patients [17]. Thiamine metabolism, expressed as the ratio of phosphorylated to unphosphorylated thiamine, is also lower in AL patients [17]. Lower levels of TDP have been found in patients with a history of poor dietary intake compared with patients with no such history [5]. Moreover, in one study, TDP levels were found to be linked to memory scores in AL, especially in patients with a history of malnutrition [5], although another study failed to replicate this result [19].

Thiamine metabolism may be disrupted by hepatic dysfunction [14], as higher thiamine T levels are found in two thirds of AL with signs of liver disease [17]. Liver disease may thus deplete the thiamine T stored in that organ and decrease the enzymatic functions required for thiamine phosphorylation [14]. An elevated level of gamma-glutamyltransferase (GGT), associated with high levels of serum glutamic oxaloacetic transaminase (SGOT) and serum glutamic pyruvic transaminase (SGPT), is regarded as a biomarker of alcohol-related liver disease [14], and have frequently been reported in AL [19,20]. Liver steatosis (asymptomatic and reversible) is observed in 20% of AL, and may progress to hepatitis or cirrhosis. The FibroMeter^®^ family of blood tests [21], an alternative to the FibroScan^®^ [22], combining platelets, the prothrombin index, alpha-2-Macroglobulin and hyaluronic acid, seems to be a relevant substitute for biopsy, as it has been found to have high diagnostic accuracy for the noninvasive diagnosis of liver fibrosis in AL [21]. Liver dysfunction is known to be responsible for neuropsychological impairments in patients with cirrhosis [23]. In AL without cirrhosis, correlations have been found between the level of GGT and mental flexibility [24], although a more recent study failed to replicate this finding [25].

Taken as a whole, these findings suggest that the origins of the cognitive heterogeneity observed in AL may lie in a combination of biological and alcoholic variables, the nature of which remains unknown. The threshold beyond which patients should be considered at risk of developing neuropsychological impairments also has yet to be determined. Finally, the identification of risk factors for severe dysfunction would allow clinicians to detect patients in need of preventive action and specific treatment. Thus, the goals of the present study were 1) to identify the potential risk factors for specific neuropsychological impairments (deficits in executive functions, visuospatial abilities, verbal episodic memory and ataxia) in recently detoxified AL, as well as those related to the severity of patients’ neuropsychological profiles (moderate or severe impairments), and 2) to determine the threshold beyond which each of these factors results in impaired neuropsychological functioning. We hypothesized that liver measures would be related to executive functions, and thiamine levels to verbal episodic memory abilities, and that the combination of the two would explain the severity of the neuropsychological profile of AL.

## Material and Methods

### Participants

Thirty AL and 15 healthy controls (HC), matched for age, education and sex (Table 1), were included in the present study. All the participants were informed about the study, approved by the local ethics committee (CPP Nord Ouest III, no. IDRCB: 2011-A00495-36), and provided their written informed consent before their inclusion (for inclusion criteria, see S1 File). The demographic and clinical characteristics of the participants, including age, education, sex ratio, Mini Mental State Examination (MMSE) score [26], Alcohol Use Disorders Identification Test (AUDIT) score [27], Beck Depression Inventory (BDI) score [28], and anxiety scores (State-Trait Anxiety Inventory (STAI) for adults, forms Y-A for state anxiety and Y-B for trait anxiety [29]) as well as their nicotine dependence level (Fagerström Test; [30]) are reported in Erreur: source de la référence non trouvée1. Although the depression, anxiety, AUDIT and nicotine dependence scores differed between the two groups, they had no effect on patients’ cognitive or motor results (verbal episodic memory, executive functions, visuospatial abilities and ataxia) as well as on the neuropsychological profiles (moderate impairment and severe impairment) (*p* > 0.05), and were therefore not included in subsequent analyses. Since depression and anxiety were exclusion criteria, it was expected not to find any relationship with patients’ neuropsychological performance.

**Table 1:** Demographic and clinical characteristics of the participants.

	Alcoholic patients	Healthy controls	*p* value
Number	30	15	
Men/women	25/5	10/5	0.20^1^
Age (years)	45.67 ± 9.57	47.93 ± 5.16	0.40
Range	33–65	37–55	
Education (years)	11.60 ± 1.69	11.60 ± 2.26	1.00
Range	9–15	9–15	
MMSE score^2^	27.10 ± 2.33	28.67 ± 1.11	0.02*
BDI score	13.53 ± 7.55	3.87 ± 2.95	< 0.001*
STAI (Y Form)			
State anxiety^2^	32.93 ± 10.75	29.07 ± 7.38	0.22
Trait anxiety^2^	44.41± 11.11	35.13 ± 6.12	0.004*
Nicotine dependence level -(Fagerström test)^2^	4.31 ± 3.63	0.20 ± 0.77	< 0.001*
AUDIT^2^	28.51 ± 6.89	3.13 ± 1.60	< 0.001*
Cushman score (during withdrawal)	4.87 ± 1.96	-	-
Alcohol use (years)	30.83 ± 10.38	-	-
Alcohol misuse (years)	18.97 ± 9.36	-	-
Daily alcohol consumption (units)	21.34 ± 10.31	-	-
Number of withdrawals	5.33 ± 9.63	-	-

*Note*. MMSE = Mini Mental State Examination; BDI = Beck Depression Inventory; STAI = Stait-Trait Anxiety Inventory; AUDIT = Alcohol Use Disorders Identification Test. Data are shown as mean ± standard deviation.

^1^ Chi^2^

^2^ One missing datum

* Significant difference between AL and HC at *p* ≤ 0.05 (*t* tests)

### Alcohol and biological variables, and nutritional measures

Blood samples were collected from all participants, either at inclusion (HC) or the day after admission to hospital (AL) (24h) to ensure that patients were free from alcohol. For all participants, blood samples were collected when they were on an empty stomach.

**Fig 1:**
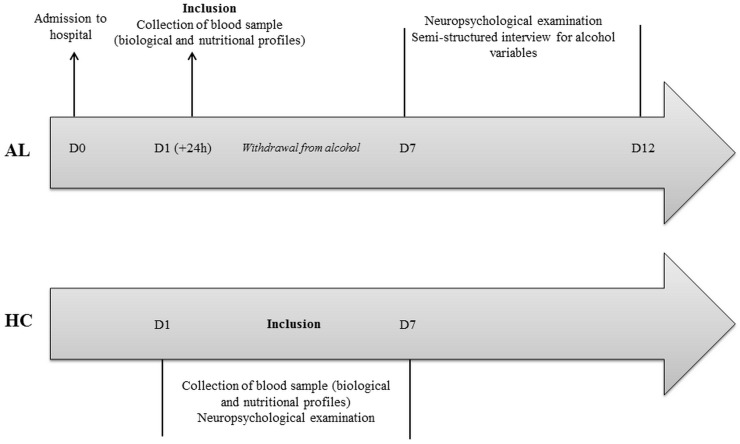
Design of the study depicting the sequence of alcohol, biological and nutritional measures as well as neuropsychological examination in alcoholic patients (AL) and healthy controls (HC).

The design of the study is depicted in Fig 1.

#### Alcohol variables

Alcohol use and misuse (in years), daily alcohol consumption (in units), number of withdrawals and Cushman score (reflecting the severity of withdrawal symptoms [31]) were selected as alcohol severity measures.

#### Malnutrition past and present

In accordance with the criteria of the French National Authority for Health (2007, www.has-sante.fr) and the ICD-10, current malnutrition was assessed by serum levels of albumin and prealbumin, body mass index (BMI; kg/m^2^) and percentage of weight lost within the previous month. Malnutrition levels ranged from 0 (no malnutrition), 1 (mild), 2 (moderate) to 3 (severe malnutrition).

Participants’ history of malnutrition was quantified by the total number of missed-meal days over their entire lifetime, through the modified version of the semi structured Lifetime Drinking History interview. An individual who reported at least 30 missed-meal days was considered to have a history of malnutrition (for criteria, see [5]. History of malnutrition was coded from 0 (no history) to 1 (positive history).

#### Thiamine

Whole-blood thiamine and phosphorylated metabolites (Thiamine T, TMP and TDP) were separated and quantified as thiochrome derivatives using liquid chromatography with fluorimetric detection [32], as previously described, with minor modifications [32,33].

In the context of their alcohol treatment, 90% of the AL included in the present study had received thiamine supplementation the day before the blood sample was collected (12 hours before): seven patients intravenously and 20 patients per os. Since thiamine supplementation could have an impact on the levels of thiamine T, TMP and TDP, only the (TDP + TMP) / (TDP + TMP + T) ratio was considered in subsequent analyses. This corresponded to the percentage of thiamine phosphorylated metabolites to all forms of thiamine in the whole-blood samples [34].

#### Liver function

Liver function was assessed on the level of GGT and the SGOT/SGPT ratio (a ratio > 1 suggested alcoholic liver disease [14]). We used the FibroMeter^®^ for the noninvasive diagnosis of alcoholic liver fibrosis [21]. This yielded a fibrosis score (ranging from 0 to 1) and a percentage of fibrosis (ranging from 0 to 100%).

### Neuropsychological examination

We administered an extensive neuropsychological battery targeting a broad range of cognitive and motor domains. *Verbal episodic memory* was assessed with the French version of the Free and Cued Selective Reminding Test (FCSRT [35]), *executive functions* with the Trail Making Test (TMT [36]) and the Modified version of the Wisconsin Card Sorting Test (MCST [37]) for flexibility, the Stroop task [38] for inhibition, the categorical verbal fluency task (animals) for memory search strategies [39] and the digit memory subtest of the MEM-III [40] for verbal working memory, *visuospatial abilities* with the Rey-Osterrieth Complex Figure (ROCF [41]) and *ataxia* with the Walk-a-Line Ataxia Battery [42]. The data collected on each test are summarized in Table A in S1 File.

The patients’ status for each of the cognitive and motor domains, as well as the severity of their neuropsychological profile (moderate or severe impairments), were computed using the method described in the S1 File.

### Statistical analyses

#### Biological and nutritional profiles of AL

The normality of the distribution of the laboratory measures for the AL and HC groups was examined using the Shapiro-Wilk test. Scores deviating from normality were log-transformed. Student *t* tests were then used to compare the two groups for measures of thiamine, nutritional status and liver function (Table 2).

**Table 2:** Laboratory and nutritional data.

Variables	Alcoholic patients	Healthy controls	*p* value
***Malnutrition***			
Albumin (g/l)	38.17 ± 3.41	42.07 ± 3.63	na
Prealbumin (g/l)	0.27 ± 0.07	0.30 ± 0.04	na
BMI (kg/m^2^)	24.55 ± 4.46	25.51 ± 4.77	na
% weight lost	2.77 ± 4.10	1.16 ± 3.16	na
Current malnutrition (0-3)	0.90 ± 0.95	0.20 ± 0.41	0.01*
History of malnutrition (0/1)	17/13	15/0	0.002*^a^^b^
***Thiamine***			
(TMP + TDP) / (TMP + TDP + T) ratio	0.87 ± 0.09	0.98 ± 0.01	< 0.001* ^a^
***Liver functions***			
GGT (U/l: log-transformed)	5.03 ± 1.50	2.98 ± 0.56	< 0.001* ^a^
Raw score	406.80 ± 636.22	23.27 ± 15.72	
SGOT (U/l; log-transformed)	4.12 ± 0.81	3.00 ± 0.34	na
Raw score	82.77 ± 63.96	21.27 ± 7.84	
SGPT (U/l; log-transformed)	3.84 ± 0.90	3.04 ± 0.52	na
Raw score	69 ± 73.30	24.00 ± 13.89	
SGOT/SGPT ratio (log-transformed)	0.27 ± 0.53	-0.04 ± 0.33	0.04*
Raw score	1.49 ± 0.74	1.00 ± 0.36	
FibroMeter^®^			
Fibrosis score (0-1; log-transformed)	0.27 ± 0.22	0.11 ± 0.07	0.008*
Raw score	0.35 ± 0.33	0.12 ± 0.09	
% fibrosis (0-100; log-transformed)	2.27 ± 0.22	1.87 ± 0.07	0.003* ^a^
Raw score	11.23 ± 8.03	6.49 ± 0.45	

* Significant difference between AL and HC at *p* < 0.05 (*t* tests).

^a^ Significant after Bonferroni correction (*p* < 0.007)

^b^ Chi^2^; data shown as numbers of participants with (1) or without (0) a history of malnutrition

*Note*. Na = not applicable. The comparison between AL and HC was not performed because these variables were used either to determine malnutrition (albumin, prealbumin, BMI and % weight lost) or to compute ratios (SGOT and SGPT).

#### Identification of risk factors for neuropsychological impairments

Four classes of variables were considered as potential risk factors for neuropsychological impairments (Table A in S1 File): alcohol severity measures, liver, thiamine and nutritional variables. In order to determine the combination of variables that best distinguished the impaired patients from the preserved ones, we ran forward logistic regressions for each cognitive or motor domain (verbal episodic memory, executive functions, visuospatial abilities, ataxia) and each profile (moderate or severe impairments). The potential risk factors were included as independent variables, and patients’ status for each of the domains and each profile as dichotomous dependent variables (0 = preserved; 1 = impaired) (for details of the selection of the independent variables, see S1 File).

For each significant factor in the final model, we performed a receiving operating characteristic (ROC) curve analysis. The goal was to determine the thresholds beyond which these measures could be regarded as risk factors for impairments in each cognitive or motor domain, and for a moderate or severe neuropsychological profile. For the biological variables that were log-transformed, we established raw cut-off values using exponential transformation.

Finally, for each step of the forward logistic regression, we examined the effect of increasing the number of variables on the value of the area under the curve (AUC), using Delong et al.’s method [43] to compare the AUC values computed at each step.

## Results

### Biological and nutritional profiles of AL

Most of the targeted laboratory measures deviated from normality and were therefore log-transformed. Compared with HC, AL had current malnutrition, impaired thiamine metabolism and liver dysfunction (Table 2).

### Neuropsychological profile of AL

63% of AL were classified as impaired on executive functions, 50% on visuospatial abilities, 40% on ataxia and 37% on verbal episodic memory as assessed in the neuropsychological examination. Regarding the severity of the neuropsychological profile, 44% of AL were classified as having at least moderate impairments, and 23% as having severe impairments.

### Identification of risk factors for neuropsychological impairments

The results of the univariate analysis are reported in Table B in S1 File, and the subsequent forward logistic regressions and cut-off scores for significant variables in Table 2.

#### Verbal episodic memory

The significant predictors for verbal episodic memory were the Cushman score and the thiamine ratio (Table 3). The ROC curves showed that AUC increased with the number of variables being considered (Figure A-A in S1 File), but the difference was not statistically significant (Table 4).

**Table 3:** Forward logistic regressions between neuropsychological profile and alcohol, biological and nutritional predictive factors.

Variables	*p*	Adjusted odds ratio 95% CI	Hosmer-Lemeshow test	Cut-off score^1^ (AUC value)
***VERBAL EPISODIC MEMORY***				
Highest Cushman score	0.04	0.60 [0.37, 1.00]	6.48, *p* = 0.60	≤ 4 (0.725)
(TMP + TDP) / (TMP + TDP + T) ratio	0.06	0.02 [0, 1.84]		≤ 0.91 (0.751)
***EXECUTIVE FUNCTIONS***				
Fibrosis score	0.02	445*10^6^ [444.*10^6^, 445*10^6^]	8.87, *p* = 0.35	> 0.22 (0.898)
***VISUOSPATIAL ABILITIES***				
None of the variables was a significant predictor				
***ATAXIA***				
% fibrosis	0.02	12.96 [9.81, 16.11]	9.50, *p* = 0.30	> 7.03 (0.863)
Alcohol misuse (years)	0.09	1.14 [0.97, 1.33]		> 13 (0.690)
Highest Cushman score	0.01	2.49 [1.21, 5.17]		> 3 (0.757)
***MODERATE IMPAIRMENT***				
Fibrosis score	0.05	39.77 [31.69, 47.85]	6.75, *p* = 0.56	> 0.22 (0.778)
(TMP + TDP) / (TMP + TDP + T) ratio	0.10	0.01 [0, 11.24]		≤ 0.89 (0.729)
***SEVERE IMPAIRMENT***				
Current malnutrition	0.02	6.84 [1.28, 36.52]	0.70, *p* = 0.99	> 0 (0.742)
(TMP + TDP) / (TMP + TDP + T) ratio	0.02	0.02 [0.00, 0.09]		≤ 0.86 (0.876)
Fibrosis score	0.13	5.65 [3.46, 7.85]		> 0.15 (0.835)

^1^ Even though biological variables were log-transformed to obtain linearity, we report the raw scores for the operational cut-off score.

**Table 4:** Effect of number of variables selected at each step of the forward logistic regressions on area under the curve (AUC).

	1 variable	2 variables	3 variables	Comparison of ROC curves*
***VERBAL EPISODIC MEMORY***	Cushman score	Cushman and thiamine ratio	na	1 vs. 2 variables: *ns*
AUC	0.706	0.739	-	
***ATAXIA***	% fibrosis	% fibrosis and misuse	% fibrosis, alcohol misuse and Cushman score	1 vs. 2 variables: *ns*; 2 vs. 3 variables: *ns*; 1 vs. 3 variables: *p* = 0.06 (*ns*)
AUC	0.806	0.806	0.889	
***MODERATE IMPAIRMENT***	Fibrosis score	Fibrosis score and thiamine ratio	na	1 vs. 2 variables: *ns*
AUC	0.729	0.808	-	
***SEVERE IMPAIRMENT***	Current malnutrition	Malnutrition and thiamine ratio	Malnutrition, thiamine ratio and fibrosis score	1 vs. 2 variables: n*s*; 2 vs. 3 variables: *ns*; 1 vs. 3 variables: *p* = 0.10 (*ns*)
AUC	0.742	0.786	0.907	

* Pairwise comparison of ROC curves

*Note*. *ns* = not significant; na = not applicable.

#### Executive functions

The only predictor for the executive profile was the fibrosis score yielded by the FibroMeter^®^ (Table 3).

#### Visuospatial abilities

None of the variables we considered were significant predictors for visuospatial abilities. When we examined the effects of the other cognitive functions, forward regression analyses revealed that executive performances were a significant predictor of visuospatial abilities (coefficient = 2.28, *p* = 0.01, odds ratio (OR) = 9.75, 95% CI [7.95, 11.55]).

#### Ataxia

The significant predictors for ataxia were the percentage of fibrosis indicated by the FibroMeter^®^, the number of years of alcohol misuse, and the Cushman score (Table 3). The ROC curves showed that AUC increased with the number of variables we considered (Figure A-B in S1 File). The AUC tended to be significantly greater (*p* = 0.06) for three variables than for one (Table 4).

#### Moderate impairment

The significant predictors for the moderate impairment profile were the fibrosis score provided by the FibroMeter^®^ and the thiamine ratio (Table 3). The ROC curves showed that AUC increased with the number of variables we considered (Figure A-C in S1 File), but the difference was not statistically significant (Table 4).

#### Severe impairment

The significant predictors for the severe impairment profile were current malnutrition, the thiamine ratio and the fibrosis score provided by the FibroMeter^®^ (Table 3). The ROC curves showed that AUC increased with the number of variables we considered (Figure A-D in S1 File). AUC tended to be significantly greater (*p* = 0.10) for three variables than for one (Table 4).

## Discussion

The present study enabled us to identify the clinical and biological factors, namely withdrawal severity, liver function, thiamine metabolism and malnutrition, that can be regarded as risk factors for neuropsychological impairments in AL. For each of these variables, we determined the threshold beyond which there was a significant risk of developing alcohol-related neuropsychological deficits.

**Fig 2:**
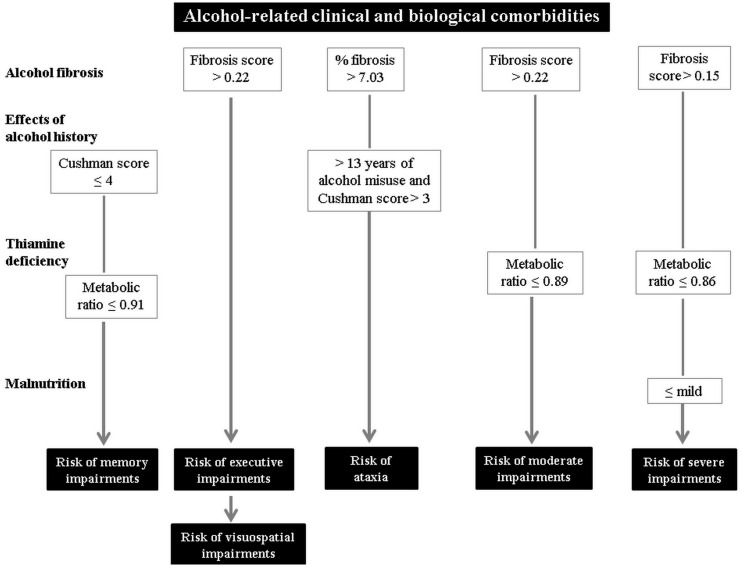
Summary of the alcohol-related clinical and biological comorbidities considered to be risk factors for impairments in verbal episodic memory, executive functions, visuospatial abilities and ataxia, as well as for moderate or severe neuropsychological impairment profiles in patients early in abstinence. The four classes of variables are listed in the left-hand column, while the other columns show the combinations of these factors giving rise to a significant risk of impairment.

In line with previous studies [19], AL had a higher GGT level and a higher SGOT/SGPT ratio than HC, as well as higher scores on the FibroMeter^®^, reflecting alcohol-related liver disease [14]. The fibrosis score of the FibroMeter^®^ was the only significant predictor of patients’ executive performances (Fig 2). A fibrosis score > 0.22, corresponding to the F1-2 stage that is considered in clinical settings to reflect biologically significant fibrosis [21], was associated with a risk of exhibiting executive impairments. In hepatic encephalopathy (HE), that represents a severe complication of hepatic dysfunction, brain abnormalities were found in frontal cortices (Lockwood et al., 2002), thalamus and cerebellum (Kril & Butterworth, 1997). These brain regions form part of the executive loop of the fronto-cerebebellar circuit (FCC), that involves the cerebellar neocortex (lobules VII, VIII, Crus I and Crus II) and the prefrontal cortex (BA 9 and 46) (Kelly and Strick 2003), contributing to set shifting and working memory (Desmond et al. 1997, 2003; Pfefferbaum et al. 2001; Seeley et al. 2007). According to Butterworth (2009), chronic liver disease leads to oxidative stress and increased brain proinflammatory cytokines, responsible for brain abnormalities observed in hepatic encephalopathy. Our data suggest that these pathophysiological mechanisms may also explain, at least partially, executive dysfunction in AL without severe hepatic encephalopathy. Our findings, in agreement with previous studies showing a relationship between mental flexibility and liver function (assessed by GGT level [24]), reinforce thus that alcohol-related liver disease has deleterious effects on executive functions. However, in the present study, neither GGT level nor SGOT/SGPT ratio was related to patients’ executive results [25]. The poor specificity of these biomarkers in the diagnosis of liver injury in alcoholism [44] could explain the absence of a relationship with cognitive functioning. Liver fibrosis, as assessed by the FibroMeter^®^, seems to be a more sensitive and specific measure when it comes to predicting the risk for associated executive impairments in AL. This finding may indicate that subclinical hepatic encephalopathy was responsible partially for these executive impairments in AL [23], even in the absence of decompensated cirrhosis. Further studies are required to examine the relationship between liver fibrosis, as assessed by the FibroScan^®^ [22], and executive functions.

Executive abilities were the only predictive factor for visuospatial performances in our sample of AL. Visuospatial deficits were not explained by any of the biological or alcohol variables included in this study (Table 3 and Fig 2). This finding is in line with the assumption that alcohol-related visuospatial impairments can be explained, at least partially, by executive dysfunctions [4]. In our study, visuospatial abilities were assessed by the ROCF copy test, involving visuospatial construction and planning, and therefore executive functions.

Liver fibrosis, assessed by the percentage of fibrosis provided by the FibroMeter^®^ (> 7.03%), was also found to be a risk factor for ataxia when it was associated with a long history of alcohol misuse and severe withdrawal symptoms (Fig 2). Ataxia, considered to reflect cerebellar abnormalities [45], is an operational criterion for the in vivo diagnosis of Wernicke’s encephalopathy [12], and has been related to more severe neuropsychological impairments in AL (6). Liver fibrosis therefore seems to be particularly deleterious to functions related to the FCC. Executive functions are supported by the executive loop of the FCC, which connects the cerebellar neocortex and prefrontal cortex, whereas motor functions (gait and balance) are supported by the motor loop, which involves the cerebellar vermis and motor cortex [46]. The association of long-term alcohol misuse, withdrawal symptoms and liver fibrosis could be responsible for more severe damage to FCC nodes, and thus to functions mediated by this brain circuit. Regarding the brain mechanisms associated with withdrawal, excessive glutamate release resulting from cessation of alcohol consumption leads to excitotoxicity and neuronal death [47]. Although glutamatergic neurons are distributed throughout the brain, the frontal cortices contain many glutamatergic pathways [48], and may therefore be more affected than other brain regions by glutamate neurotoxicity arising from alcohol withdrawal. Thus, the moderate-to-severe symptoms reflecting the neurotoxicity of withdrawal, as assessed by the Cushman score, may be related to cognitive and motor deficits sustained by the frontal cortices. This result should prompt researchers to investigate the effects not only of chronic and excessive alcohol consumption, but also of the neurotoxicity of withdrawal on neuropsychological functioning in AL.

Although 90% of the patients included in the present study benefited from thiamine supplementation before the blood draw (12 hours before), an exploratory between-group analysis suggested that the metabolic ratio did not differ between the three subgroups of patients (no supplementation (n = 3; mean = 0.95 ±0.04), intra-venous (n = 7; mean = 0.82 ±0.08) and per os (n = 20; mean = 0.88 ±0.09; data not shown, no difference with non-parametric comparisons), as previously reported with a different ratio [17]. Even though these findings should be interpreted with caution given the small sample size, we can postulate that it is unlikely that supplementation biased our results. Despite supplementation and in agreement with a previous study [17], AL had an abnormal thiamine metabolism, expressed by a lower phosphorylation ratio compared with HC, resulting from elevated T level but no elevated level of TDP. These findings emphasize the need to investigate not only the biologically active form of thiamine (TDP) but also its metabolism. Impaired thiamine metabolism, which may be related to liver dysfunction [14,17], could explain why thiamine supplementation seems inefficient in some patients. The combination of impaired thiamine metabolism and mild symptoms of alcohol withdrawal may be a risk for alcohol-related episodic memory impairments (Fig 1). This finding is in line with a previous study that showed that a lower TDP level is selectively related to poorer memory performances [5]. As the association of thiamine deficiency and long-term chronic alcoholism is the main etiology of Korsakoff’s syndrome [7], patients with impaired thiamine metabolism and a long history of alcohol misuse may be at risk of neurological complications.

Contrary to what we found for ataxia, a low Cushman score (≤ 4) during withdrawal from alcohol was associated with episodic memory deficits. In clinical practice, this cut-off score is the threshold below which the symptoms of alcohol withdrawal are considered to be under control [31]. One possible explanation is that patients with a low Cushman score had received more benzodiazepine (BZD) medication during detoxification, which is supposed to limit the severity of withdrawal symptoms. The deleterious effects of BZD on episodic memory functioning, well described in the literature (for a review, see [49]), could explain the differential role of the Cushman score as a risk factor for executive and episodic memory impairments. Contrary to previous studies, alcohol severity measures such as recent alcohol drinking [8,9], duration of alcohol dependence [3,8], and number of withdrawal periods [10], were not predictive of neuropsychological impairments in AL.

The combination of these alcohol-related clinical and biological comorbidities regarded as risk factors for specific cognitive and motor impairments in AL was associated with the severity of the neuropsychological profile. Poor thiamine metabolism and the presence of liver fibrosis were significant risk factors for moderate impairment (Fig 2). Our criterion for moderate neuropsychological impairment was the presence of at least two affected cognitive domains, potentially including episodic memory and executive impairments, in which abnormal thiamine metabolism and liver function were selectively involved. Thus, the combination of these two biological comorbidities in AL could predict the risk of multiple cognitive impairments and be regarded as a factor for cognitive heterogeneity in alcoholism. Finally, the neuropsychological profile of AL seemed to be exacerbated by current malnutrition. Low thiamine metabolism and liver fibrosis associated with malnutrition, diagnosed using the HAS (2007, www.has-sante.fr) and ICD-10 criteria, were predictive of the risk for severe alcohol-related neuropsychological impairment (Fig 2). Malnutrition is an operational criterion for the in vivo diagnosis of Wernicke’s encephalopathy [12], and has previously been put forward to explain the cognitive heterogeneity of AL [5]. Our findings suggest that patients with disrupted thiamine metabolism, potentially resulting from both malnutrition and liver dysfunction [14,17], should receive close clinical attention, as they are at particular risk of severe neuropsychological impairments and may potentially have signs of Wernicke’s encephalopathy [5].

The relatively small sample size of the groups we included in the present study (thirty AL and 15 HC) may be considered as a limitation. However, it is very challenging to include carefully selected controls, matched for age, education and sex with AL, and without comorbidities that would affect blood measures, especially for such as extensive neuropsychological and biological assessment.

To conclude, this study revealed that alcohol-related clinical (severity of withdrawal) and biological (liver dysfunction, malnutrition, and impaired thiamine metabolism) comorbidities are associated with specific risks for cognitive and motor impairments in AL. Liver fibrosis was associated with a risk of impairment in functions mediated by the FCC (executive functions, gait and balance), while altered thiamine metabolism was related to episodic memory deficits. The pattern of alcohol consumption made a limited contribution to the neuropsychological profile of AL. By contrast, the severity of alcohol withdrawal symptoms had differing effects, depending on which function we considered. Overall, the combination of these comorbidities was found to be associated with the severity of the neuropsychological profile. Thus, malnutrition, as well as impaired liver function and thiamine metabolism, may be responsible for the heterogeneity of alcohol-related neuropsychological deficits. Moreover, the present study provided thresholds for each of the clinical and biological variables, beyond which the risk of developing neuropsychological impairments can be considered significant. These thresholds led to the establishment of operational criteria that can now be used in a clinical setting. If these findings are associated with a screening tool for alcohol-related neuropsychological impairments, such as the BEARNI [50], clinicians will be able to identify patients at risk of severe impairment and needing preventive action and specific care, including nutritional and hepatic treatment.

## Supporting Information

S1 File Figure A: Comparison of ROC curves according to number of variables selected at each step of the forward logistic regression. A: Comparison of ROC curves for one variable (highest Cushman score) and two variables (highest Cushman score and (TMP + TDP) / (TMP + TDP + T) ratio) regarded as risk factors for episodic memory impairments. B: Comparison of ROC curves for one variable (%fibrosis), two variables (%fibrosis and alcohol misuse) and three variables (%fibrosis, alcohol misuse and highest Cushman score) regarded as risk factors for ataxia impairments. C: Comparison of ROC curves for one variable (fibrosis score) and two variables (fibrosis score and (TMP + TDP) / (TMP + TDP + T) ratio) considered as risk factors for moderate impairments. D: Comparison of ROC curves for one variable (current malnutrition), two variables (current malnutrition and (TMP + TDP) / (TMP + TDP + T) ratio) and three variables (current malnutrition, (TMP + TDP) / (TMP + TDP + T) ratio and fibrosis score) considered as risk factors for severe impairments. Table A in S1 File: Summary of the cognitive and motor scores used in the analyses. Table B in S1 File: Results of the univariate analyses between each cognitive or motor domain and severity profile measured in the neuropsychological examination and each potential predictive variable.
(DOCX)
