# Supplementary material for: Clinical and Biological Risk Factors for Neuropsychological Impairment in Alcohol Use Disorder
Source: PLoS One. 2016 Sep 12;11(9):e0159616. doi: 10.1371/journal.pone.0159616 (PMC5019388; doi:10.1371/journal.pone.0159616)
Supplement: S1 File — Figure A: Comparison of ROC curves according to number of variables selected at each step of the forward logistic regression. A: Comparison of ROC curves for one variable (highest Cushman score) and two variables (highest Cushman score and (TMP + TDP) / (TMP + TDP + T) ratio) regarded as risk factors for episodic memory impairments. B: Comparison of ROC curves for one variable (%fibrosis), two variables (%fibrosis and alcohol misuse) and three variables (%fibrosis, alcohol misuse and highest Cushman score) regarded as risk factors for ataxia impairments. C: Comparison of ROC curves for one variable (fibrosis score) and two variables (fibrosis score and (TMP + TDP) / (TMP + TDP + T) ratio) considered as risk factors for moderate impairments. D: Comparison of ROC curves for one variable (current malnutrition), two variables (current malnutrition and (TMP + TDP) / (TMP + TDP + T) ratio) and three variables (current malnutrition, (TMP + TDP) / (TMP + TDP + T) ratio and fibrosis score) considered as risk factors for severe impairments. Table A in S1 File: Summary of the cognitive and motor scores used in the analyses. Table B in S1 File: Results of the univariate analyses between each cognitive or motor domain and severity profile measured in the neuropsychological examination and each potential predictive variable. (DOCX) [file pone.0159616.s001.docx]

Table A: Summary of the cognitive and motor scores used in the analyses

| **Domains** | **Tests** | **Retained scores** |
| --- | --- | --- |
| ***Verbal episodic memory*** | French version of the FCSRT | Encoding (sum of three total recall tests)  Learning and retrieval (sum of three free recall tests)  Long-term retrieval (delayed free recall)  Long-term consolidation (delayed total recall) |
| ***Executive functions*** | Flexibility  Inhibition  Memory search strategies  Verbal working memory | TMT Parts B-A (time)  MCST (no. perseverations)  Stroop Interference - Naming (time)  Fluency task (animal category; no. correct responses)  Standard score of the MEM III digit memory subtest |
| **Visuospatial abilities** | ROCF | Copy accuracy /36  Type of copy (I-V) |
| ***Ataxia*** | Walk-a-Line Ataxia Battery | Stand in Romberg position (feet placed heel to toe) (two 60-s trials; eyes open)  Stand in Romberg position (feet placed heel to toe) (two 60-s trials; eyes closed)  Walk heel-to-toe (two 10-step trials, eyes open)  Walk heel-to-toe (two 10-step trials, eyes closed) |

*Note.* FCSRT = Free and Cued Selective Reminding Test; MCST = Modified Card Sorting Test; TMT B-A: Trail Making Test Part A minus Part B; ROCF: Rey‑Osterrieth Complex Figure.

Table B: Results of the univariate analyses between each cognitive or motor domain and severity profile measured in the neuropsychological examination and each potential predictive variable

| **Variables** | **Verbal episodic memory** | **Executive function** | **Visuospatial abilities** | **Ataxia** | **Moderate impairment** | **Severe impairment** |
| --- | --- | --- | --- | --- | --- | --- |
| ***Alcohol variables*** | | | | | | |
| Cushman score | 0.06* | 0.70 | 0.49 | 0.02* | 0.67 | 0.13* |
| Alcohol use | 0.10* | 0.58 | 0.20 | 0.41 | 0.51 | 0.52 |
| Alcohol misuse | 0.76 | 0.56 | 0.60 | 0.07* | 0.74 | 0.18 |
| Daily alcohol consumption | 0.89 | 0.71 | 0.19 | 0.22 | 0.63 | 0.48 |
| Number of withdrawals | 0.81 | 0.17 | 0.34 | 0.25 | 0.14 | 0.15 |
| ***Liver function*** | | | | | | |
| Level of GGT | 0.68 | 0.04* | 0.40 | 0.02* | 0.31 | 0.01* |
| TGO/TGP ratio | 0.15 | 0.60 | 0.25 | 0.13 | 0.56 | 0.53 |
| Fibrosis score | 0.04* | 0.02* | 0.41 | 0.04* | 0.02* | 0.01* |
| % fibrosis | 0.17 | 0.01* | 0.89 | 0.03* | 0.17 | 0.02* |
| ***Thiamine*** | | | | | | |
| (TMP + TDP) / (TMP + TDP + T) ratio | 0.07* | 0.08* | 0.29 | 0.16 | 0.04* | 0.03* |
| ***Malnutrition*** | | | | | | |
| Current malnutrition | 0.40 | 0.34 | 0.97 | 0.22 | 0.37 | 0.01* |
| History of malnutrition | 0.86 | 0.27 | 0.35 | 0.04* | 0.78 | 0.10* |

* Significant variables at *p* ≤ 0.10 retained in the subsequent forward stepwise logistic regression (in gray)


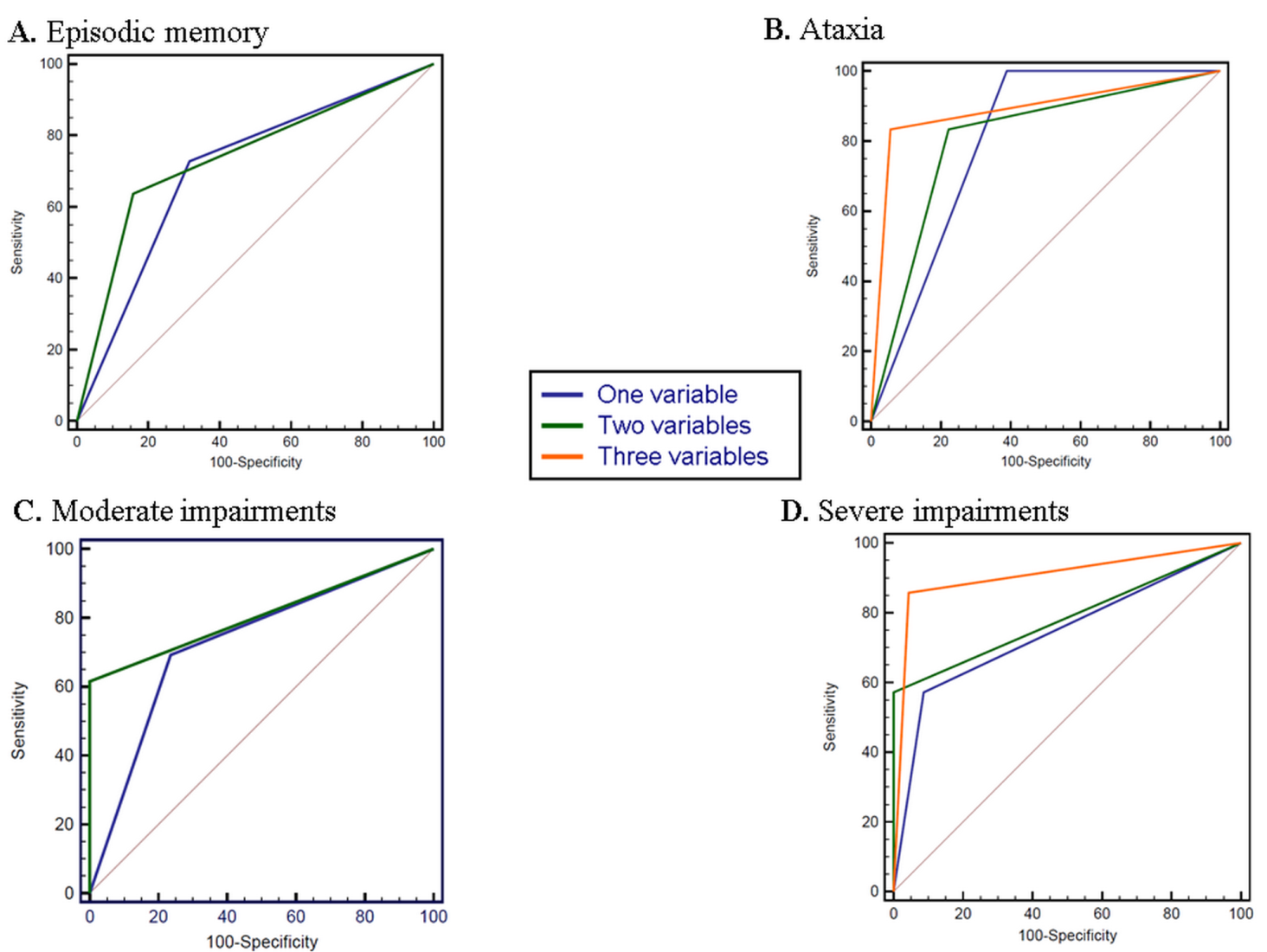


Figure A: Comparison of ROC curves according to number of variables selected at each step of the forward logistic regression.

A: Comparison of ROC curves for one variable (highest Cushman score) and two variables (highest Cushman score and (TMP + TDP) / (TMP + TDP + T) ratio) regarded as risk factors for episodic memory impairments.

B: Comparison of ROC curves for one variable (%fibrosis), two variables (%fibrosis and alcohol misuse) and three variables (%fibrosis, alcohol misuse and highest Cushman score) regarded as risk factors for ataxia impairments.

C: Comparison of ROC curves for one variable (fibrosis score) and two variables (fibrosis score and (TMP + TDP) / (TMP + TDP + T) ratio) considered as risk factors for moderate impairments.

D: Comparison of ROC curves for one variable (current malnutrition), two variables (current malnutrition and (TMP + TDP) / (TMP + TDP + T) ratio) and three variables (current malnutrition, (TMP + TDP) / (TMP + TDP + T) ratio and fibrosis score) considered as risk factors for severe impairments.

**Participant inclusion criteria**

Alcoholic patients (AL) were recruited by clinicians while they were receiving withdrawal treatment as inpatients at Caen University Hospital. All patients met the DSM-IV criteria for alcohol dependence for at least 5 years. They completed the Alcohol Use Disorders Identification Test (AUDIT) (28) and a modified version of the semi-structured Lifetime Drinking History interview, where measures include the duration of alcohol use (in years), alcohol misuse (in years), number of withdrawals, and daily alcohol consumption over the previous month (in units, a standard drink corresponding to a beverage containing 10 g of pure alcohol) (Table 1). Although patients were early in abstinence when they underwent the neuropsychological examination, none of them displayed the physical symptoms of alcohol withdrawal, as assessed by the Cushman scale. The highest Cushman score during withdrawal was recorded and is reported in Table 1.

AL were compared with 15 healthy controls (HC). All the HC were interviewed with the AUDIT questionnaire to ensure that they did not meet the criteria for alcohol abuse or dependence (AUDIT < 7 for men and < 6 for women). None of the HC had an MMSE score below the cut-off score of 26 (24).

None of the participants had a history of neurological pathologies, endocrine or infectious diseases (diabetes, HIV or hepatitis confirmed by blood analysis), mental illness, depression (Beck Depression Inventory; BDI-II), or other forms of substance misuse or dependence (except tobacco). Furthermore, none of them were under psychotropic medication that might have had an effect on their cognitive functioning.

**Participants’ neuropsychological profiles**

The neuropsychological profiles of healthy controls (HC) and alcoholic patients (AL) were determined using the method described in (40). Scores on the neuropsychological battery were used to determine the cognitive or motor domains impaired in AL which, in turn, were used to determine the severity of the neuropsychological profile (see below for details). First of all, the normality of the distribution of all neuropsychological scores in HC and AL was examined using kurtosis, skewness and the Shapiro‑Wilk test. Scores deviating from normality were log-transformed. The potential effects of age, education and sex were then tested in HC with linear regression analysis (forward stepwise), and only significant factors were retained. Regression coefficients computed in HC were used to compute standardized residuals in the two groups (i.e., *z* scores, with poor performances corresponding to negative *z* scores), to take the effects of demographic factors into account. Each participant’s *z* scores (Table S1) were classified as preserved (0 when > -1.65) or impaired (1 when ≤ -1.65). The status of AL for each cognitive or motor domain was then determined as follows:

- Verbal episodic memory: at least two impaired *z* scores. This criterion was chosen so that the prevalence of AL with verbal memory deficits in our study matched that reported in previous studies (1);
- Executive functions: at least two impaired *z* scores;
- Visuospatial abilities: one impaired *z* score for copy accuracy and/or type of copy > II (42);
- Ataxia: at least two impaired *z* scores (6).

Finally, the severity of the neuropsychological profiles was specified according to the following criteria:

- Moderate impairment: at least two affected cognitive domains (out of verbal episodic memory, executive functions and visuospatial abilities);
- Severe impairment: at least two affected cognitive domains, associated with ataxia. This criterion was chosen because ataxia was identified as an operational criterion by Caine et al. (13) for the in vivo diagnosis of Wernicke’s encephalopathy, and is reportedly related to more severe neuropsychological impairments in AL (6).

**Statistical analyses: identification of risk factors for neuropsychological impairments**

First of all, we ran univariate analyses (simple logistic regressions) to assess the relationships between the status for each cognitive or motor domain and the two severity profiles, and each of the potential risk factors (Table S1 in Supplemental Information). Only variables significant at *p* ≤ 0.10 were entered in the logistic regressions to determine which variables remained independent predictors (*p* < 0.05) and best distinguished impaired patients from preserved ones. The final models were checked using the Hosmer-Lemeshow goodness-of-fit test.

To avoid multicollinearity in the regression models, correlations between the potential explanatory variables were examined with pairwise correlations (Pearson’s *r*). Some of them were moderately correlated but none of the correlation coefficients reached the value of > 0.6 that is usually considered to suggest multicollinearity.
